# The impact of early oral anticancer medication monitoring through electronic patient assessments

**DOI:** 10.1093/ajhp/zxag070

**Published:** 2026-03-07

**Authors:** Brooke Looney, Tiffany Bui, Josh DeClercq, Kristen Whelchel, Leena Choi, Autumn Zuckerman

**Affiliations:** Vanderbilt Specialty Pharmacy, Vanderbilt University Medical Center, Nashville, TN, USA; Northwestern Medicine Specialty Pharmacy, Northwestern Medicine, Chicago, IL, USA; Department of Biostatistics, Vanderbilt University Medical Center, Nashville, TN, USA; Vanderbilt Specialty Pharmacy, Vanderbilt University Medical Center, Nashville, TN, USA; Department of Biostatistics, Vanderbilt University Medical Center, Nashville, TN, USA; Vanderbilt Specialty Pharmacy, Vanderbilt University Medical Center, Nashville, TN, USA

**Keywords:** cancer, medication monitoring, patient portals, specialty pharmacy, technology

## Abstract

**Purpose:**

This study evaluated the effectiveness of electronic early oral anticancer medication monitoring questionnaires in identifying adverse effects (AEs) requiring pharmacist intervention among patients initiated on oral anticancer therapy.

**Methods:**

A prospective randomized study was conducted within an integrated health system specialty pharmacy. Patients were excluded if they never started treatment, discontinued oral anticancer therapy within 14 days, were not new to therapy, or had a hematologic condition. Stratified randomization by age (≥60 years versus <60 years) and sex was performed to assign patients to intervention or usual care. Intervention patients received an electronic questionnaire 7 to 14 days after initial counseling. Outcomes included time to first AE-related pharmacist intervention and intervention frequency within 45 days and dose modifications, healthcare utilization, and treatment modifications within 90 days. Per-protocol analysis compared outcomes among questionnaire responders and patients assigned to usual care. Cox proportional hazard (time to intervention) and ordinal logistic regression (intervention number) models adjusted for age, sex, race, diagnosis, Charlson comorbidity index score, and medication type.

**Results:**

Of the 446 patients randomized, 388 were included in the analysis (183 in the intervention cohort and 205 in the usual care cohort). The questionnaire response rate was 47%. In the intent-to-treat analysis, no significant differences were observed in time to intervention (hazard ratio [HR], 1.2; *P* = 0.360) or intervention frequency (odds ratio [OR], 1.2; *P* = 0.409). Per-protocol analysis (n = 291) showed responders had significantly faster time to intervention (HR, 2.4; *P* < 0.001), more interventions (OR, 2.7; *P* < 0.001) and lower healthcare utilization (OR, 0.4; 95% CI, 0.2-0.9; *P* = 0.023).

**Conclusion:**

Electronic monitoring resulted in earlier and more frequent interventions and less healthcare utilization among questionnaire responders.

Key Points

- Almost half of patients responded to an electronic monitoring questionnaire sent through their electronic health portal for early oral anticancer monitoring.
- Patients who responded to the questionnaire had earlier and more frequent adverse event–related pharmacist interventions and lower healthcare utilization.
- Electronic questionnaires are likely beneficial for oral anticancer monitoring but may require additional patient counseling to improve uptake; patient communication preferences should be considered.

The growing number of oral anticancer medications used as preferred cancer treatments has emphasized the role specialty pharmacists play in managing oncology treatments.^1,2^ Between 2013 and 2017, the number of oral anticancer medications dispensed through Medicare Part D increased by more than 1 million, accounting for 8,164,833 prescriptions in 2017.^3^ Though these therapies offer effective and more convenient treatment options, they present new challenges regarding medication access and monitoring.^4^ Adverse effects (AEs) such as diarrhea, nausea and vomiting, and rash may occur early in therapy (often within the first 30 days) and may limit a patient’s ability to adhere to therapy or continue a specific dose or medication.^5-7^ Nonadherence can result in treatment ineffectiveness, and early treatment discontinuation contributes to significant levels of waste.^8,9^ Identifying and managing AEs early can help patients continue on treatment at higher doses.^10,11^

Previous research has demonstrated that specialty pharmacist involvement in anticancer medication monitoring can increase adherence,^12^ improve patient understanding of new oral anticancer treatments,^1^ reduce patient costs,^13,14^ and result in faster time to medication receipt^12,15^ and high patient satisfaction.^16^ Integrated specialty pharmacists in oncology clinics are involved throughout the patient journey, including assisting with medication selection, ensuring affordable access to treatment, monitoring and optimizing therapy to ensure therapeutic benefits are realized, and reducing medication waste.^17-19^ The Hematology/Oncology Pharmacy Association (HOPA) “Best Practices for the Management of Oral Oncolytic Therapy” recommends monitoring patients initiated on oral therapy for symptoms and adherence between 7 and 14 days following treatment initiation.^20^ This frequency of patient monitoring can pose a burden on clinic and/or pharmacy staff who may already be experiencing a high rate of burnout.^21-23^ Additionally, tailored monitoring protocols, wherein pharmacists contact patients at specific time points when AEs are most likely to occur, have proven useful for some anticancer medications^10,11^ but not others.^19^ Methods to identify and address early AEs or nonadherence without adding significant burden to the pharmacist have not been adequately explored and are needed.

Electronic health record (EHR) patient portals are an increasingly common method of patient communication.^24^ A study from 2023 reported that patient portal use rose from 31% in 2014 to 68% in 2022, with a notable 46% increase between 2020 and 2022.^25^ Though the potential benefits of this communication platform are numerous, the evidence supporting electronic patient portal utilization and any resulting clinical impact is limited.^26,27^ Using technology, specifically an automatic assessment through the patient portal, could offer a convenient solution for early patient monitoring while not significantly adding to clinic workload.

As part of the current prospective randomized study described herein, a previous publication described the development, implementation, and implementation outcomes of an electronic early treatment assessment and monitoring (eTEAM) questionnaire for patients initiated on oral anticancer therapy.^28^ The previous analysis, guided by the RE-AIM (Reach, Effectiveness, Adoption, Implementation, and Maintenance) framework, demonstrated that the eTEAM questionnaire, sent through the EHR patient portal, was well received by patients and pharmacists. The response rate was 47%, and patient responses enabled pharmacists to identify AEs early and address adherence concerns. Pharmacists reported that the questionnaire was appealing, feasible, and positively impacted their practice and patient care without significantly increasing workload. The previous findings support the utility of the eTEAM questionnaire as a viable monitoring strategy and provide the foundation for evaluating its impact on patient care.

In this analysis we present findings comparing the eTEAM questionnaire monitoring method to usual care. Therefore, this study fills a gap in the literature by evaluating whether a proactive patient assessment sent via the electronic patient portal is beneficial in early anticancer treatment monitoring.

## Objective(s)

The objective of this study was to evaluate if sending an eTEAM questionnaire to patients initiating oral anticancer therapy improves identification of AEs that necessitate a pharmacist intervention or affects early treatment outcomes.

The study aims were (1) to evaluate differences in timing and frequency of AE identification resulting in a pharmacist intervention during the first 45 days from initial prescription dispense in patients receiving an eTEAM questionnaire within 14 days from initial dispense, as compared to those receiving usual care refill assessments at the time of refill delivery scheduling; and (2) to evaluate the extended impact (within the first 90 days of treatment) of early patient monitoring using an automated electronic patient assessment.

## Methods

### Study design

A single-center, randomized, pragmatic cohort study was completed to evaluate the impact of using an eTEAM questionnaire on pharmacist interventions for AEs in patients starting oral anticancer therapy from August 15, 2023, to February 29, 2024. A stratified randomization between intervention versus usual care at a 1:1 ratio was performed within each stratum of age (≥60 years vs <60 years) and sex using a tool designed by the institution’s Research HealthIT Department. Randomization was stratified by age and sex because these demographic factors were anticipated to influence patients’ likelihood of responding to the electronic questionnaire based on previous literature.^29-31^ In contrast, we did not expect medication type or cancer diagnosis to significantly affect response behavior and thus did not include them in the stratification. This study received approval from the institution’s institutional review board, and a waiver of consent was granted. eTEAM questionnaire implementation outcomes including reach, adoption, implementation, and maintenance were reported previously.^28^

### Setting

The study was conducted at a large academic health system in the southeastern United States. The institution has an integrated health-system specialty pharmacy (HSSP); at the time of the study 4 pharmacists and 2 pharmacy technicians were embedded into the outpatient specialty clinic. Specialty pharmacists have been integrated into the oncology clinic since 2013 and are key multidisciplinary team members. When patients are prescribed an oral anticancer medication, a pharmacist and a pharmacy technician are made aware of the treatment plan and assist the patient with medication access and affordability, including completing insurance authorization requirements and obtaining financial assistance if needed. If the integrated HSSP is contracted to fill the medication prescription for the patient and the patient chooses to use the pharmacy, pharmacists provide detailed counseling in person, by phone, or via virtual conference call. During monthly prescription refill calls, pharmacists follow up on patient-reported AEs, medication list changes, adherence, administration, and access concerns. Additionally, pharmacists perform reassessments to evaluate the continued safety and appropriateness of therapy annually. Each month the integrated specialty pharmacy manages approximately 1,050 active patients filling an oral anticancer medication prescription with the pharmacy, including about 100 patients with new-start prescriptions from the institution’s Cancer Center. Approximately 85% of patients filling oral anticancer medication prescriptions through the specialty pharmacy have an active EHR patient portal account wherein they can communicate with the provider and healthcare team. Patients can also call into the clinic or the pharmacy at any time, as they are given the number for the direct phone line to the pharmacist at the time of initial counseling.

Other healthcare team members, including the prescribing provider, do not have standardized patient outreach protocols for use in between clinic visits.

### Pharmacist interventions

Pharmacists document AEs and pharmacist interventions in the specialty pharmacy patient management database using a pharmacist intervention assessment. The assessment categorizes the reason for the intervention, actions taken by the pharmacist, and the outcome of the intervention. Reasons for intervention include an AE, adherence issues, and issues related to medication reconciliation/interaction, healthcare use, medication administration, and care coordination. Actions taken by the pharmacist (pharmacist interventions) could include providing additional patient education, recommending supportive nonprescription or prescription medications, coordination of care for appointments or laboratory monitoring, and escalating issues to the prescriber with a recommendation to interrupt, change, or stop therapy. Potential outcomes of the actions include the identified issue being resolved, adjunct therapy being started, medication being held and follow-up care scheduled, or a medication dose being adjusted. AEs and notable pharmacist interventions are communicated to the provider, and therapy recommendations or changes are discussed.

### Study population

The study included patients meeting the following criteria: prescribed new oral anticancer medication (defined as the same oral anticancer agent not being prescribed in the last year), at least one fill of the new oral anticancer medication through the institution's integrated specialty pharmacy, active patient portal account, age of ≥18 years, and not prescribed any of 6 specific oral anticancer medications (sorafenib, regorafenib, olaparib, niraparib, rucaparib, and talazoparib). These 6 medications were excluded as the institution’s specialty pharmacy had previously implemented early tailored monitoring protocols that differ from the usual-care call schedule.^10,11,19^ On average, prescriptions for these 6 medications were filled for only 9 patients monthly; therefore, the impact of excluding these patients on study generalizability was anticipated to be low. Patients were excluded if they were prescribed treatment for a hematologic malignancy, discontinued treatment within 14 days of initiation, or never initiated medication use.

### Randomization procedures

Randomization to the intervention or usual care cohort occurred when initial medication counseling documentation was saved in the EHR by the pharmacist. A randomization tool within the EHR assessed whether or not the patient met inclusion criteria parameters. Patients meeting inclusion criteria were randomized to the intervention or usual care cohort. Patients assigned to the intervention cohort auto-populated in an EHR report 7 days after the initial medication counseling documentation was saved in the EHR. A specialty pharmacist reviewed the report of intervention patients twice a week. At that time, the pharmacist assessed the medication start date. If the patient had started the medication 7 to 14 days prior to review, the pharmacist sent the patient an eTEAM questionnaire from the report. If a patient had not started treatment in the past 7 to 14 days, the questionnaire was held until the next scheduled pharmacist review. Patients were included on the report until 14 days after initial counseling documentation or questionnaire assignment. If the questionnaire was not sent within 14 days, the patient dropped off the report and was included in the usual care cohort.

### Usual care cohort

Usual care for patients using the integrated HSSP involved monitoring patients based on medication-specific clinical protocols. Patients were contacted at least monthly approximately 5 to 7 days before a refill due date (typically 21-28 days after initiation of a 30-day supply of medication), and adherence, AEs, and the need for additional follow-up with the specialty pharmacist were assessed by a pharmacy technician (this was termed “refill assessment”). Pharmacist interventions were provided to patients in the usual care cohort based on patient responses to the refill assessment or if patients contacted the clinic or pharmacist directly with a medication-related issue. There was not a standard early monitoring assessment (ie, an assessment within 7 to 14 days after medication initiation) for the usual care patients.

### Intervention cohort

**Figure 1.**
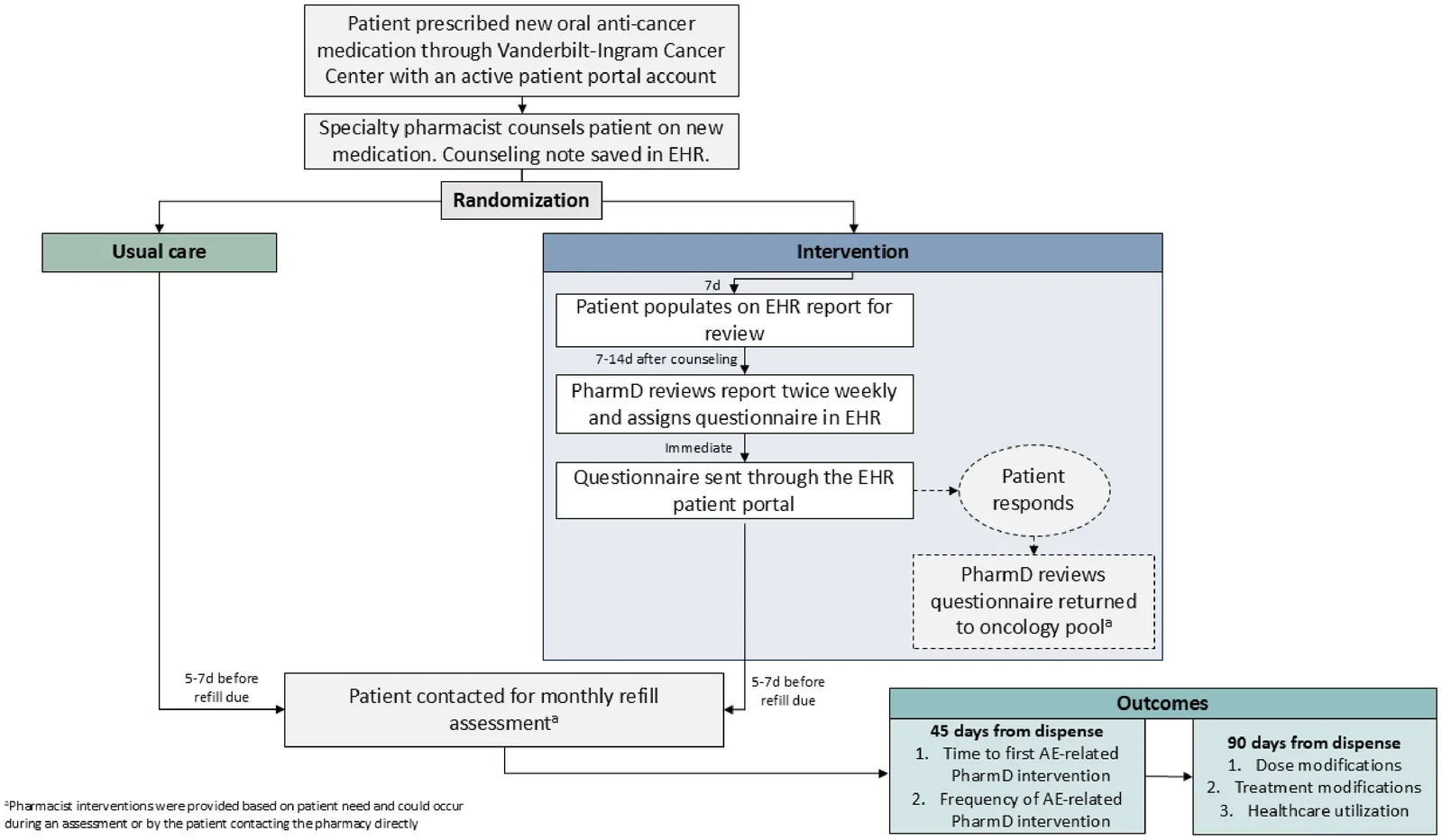
The diagram depicts study workflow from the time a patient was prescribed a new oral anticancer medication through randomization to either the intervention or usual care, as well as contact points within the first month of treatment. Study outcomes at 45 and 90 days are shown. AE indicates adverse effect; EHR, electronic health record; PharmD, pharmacist.

Pharmacists sent the eTEAM questionnaire to patients via the patient portal by clicking a button on the report of intervention patients. The questionnaire automatically populated in the patient’s portal and was available for patients to complete for up to 7 days. Patients randomized to the intervention also received the regularly scheduled refill calls 5 to 7 days prior to their refill being due (Figure 1).

As previously described, the eTEAM questionnaire consisted of up to 11 questions that branched based on patient response, including if and when the patient started their medication, if they had missed any doses, whether they were experiencing any AEs, how they managed the AEs, if they had questions for a pharmacist, and preferred contact method.^28^ The eTEAM questionnaire can be found in eFigure 1.

Completed questionnaires populated in the oncology specialty pharmacist message basket within the EHR under the heading “Patient Questionnaires.” The message basket is a working queue for specialty pharmacists to receive and send messages to patients and healthcare providers. The specialty pharmacists reviewed patient responses and intervened if the patient reported adherence issues, AEs, or designated they would like to be contacted by the specialty pharmacist. If the pharmacist intervened, a pharmacist intervention assessment documentation form was completed (as described above).

### Analysis datasets

For data analysis, we used 2 sets: the intention-to-treat (ITT) and per-protocol (PP) datasets. In this study, “per protocol” was defined as responding to the survey questionnaires sent to patients. The ITT set included all patients as randomized, regardless of protocol adherence, so that the intervention group comprised both responders and nonresponders. In contrast, the PP set included all patients randomized to the usual care arm but only those in the intervention arm who responded to the questionnaire.

### Outcomes

The primary outcome was time to first pharmacist intervention related to AEs within 45 days of medication dispensing. Only interventions related to an AE were included, though a single intervention could have multiple reasons. The 45-day window allowed for all patients to have at least one refill assessment. The secondary outcome was the number of pharmacist interventions per patient, treated as an ordinal outcome, based on patient-reported AEs within the first 45 days of the initial oral anticancer medication dispense. The exploratory outcomes were composite measures derived from 3 clinical domains: (1) dose modification (the sum of dose interruptions and dose changes within 90 days of the initial dispense); (2) healthcare utilization (the sum of hospitalizations and emergency department visits within 90 days of the initial dispense); and (3) treatment modification (the sum of discontinuation and medication change within 90 days of the initial dispense). All composite outcomes were treated as ordinal outcomes. Study outcomes are summarized in Figure 1. Data was stored in REDCap (Research Electronic Data Capture) tools hosted within the institution.^32,33^

### Statistical analysis

For descriptive statistics, continuous variables are presented as medians with interquartile range (IQR), and categorical variables are presented as frequencies and percentages. Descriptive statistics were used to summarize the study samples including the outcomes. For the primary outcome analysis, a Cox proportional hazard model was used to examine the difference in time to pharmacist interventions in the intervention and usual care cohorts. The analysis adjusted for selected a priori covariates: age group, sex, Charlson comorbidity index (CCI) score, and medication type (antineoplastic agents or antiandrogens/hormone receptor antagonists). For the secondary outcome analysis, ordinal logistic regression was used to assess the association between the number of interventions and the covariates listed above. For each exploratory outcome, ordinal logistic regression analysis was performed, with controlling for study assignment, age, sex, and CCI score. All analyses were conducted using R version 4.4.0 (R Foundation for Statistical Computing, Vienna, Austria), and *P* values less than 0.05 were considered statistically significant.

## Results

### Sample

Of the 446 patients randomized, 58 were excluded due to medication being prescribed for hematologic malignancy (48%), not starting medication use within 14 days (29%), or not being new to therapy (22%). The allocation for the remaining 388 patients was 183 to the intervention cohort and 205 to the usual care cohort. Although differences in baseline patient characteristics were observed by chance, the 2 cohorts were generally well balanced, particularly for the key stratification factors: age ≥60 years (68% in the intervention cohort vs 70% in the usual care cohort) and sex (61% male in the intervention cohort vs 57% in the usual care cohort). Genitourinary cancers accounted for 46% and 40% of diagnoses in the intervention and usual care groups, respectively. Over half of patients in both cohorts had stage IV disease (59% of intervention and 69% of usual care patients). Antineoplastic agents were most commonly used drug class (62% of intervention and 71% of usual care patients), with slightly less than half of treatment regimens being first line (40% of intervention and 48% of usual care patients) (Table 1). Among the 182 intervention patients, the questionnaire response rate was 47% (n = 86). Characteristics of the study groups are shown in eTable 1.

**Table 1.** Demographic and Clinical Data for Study Cohorts

Characteristic	Intervention (n = 183)	Usual care (n = 205)
Sex (male), No. (%)	112 (61)	116 (57)
Age group (≥60 years), No. (%)	124 (68)	144 (70)
CCI score, median (IQR)	7 (5-9)	8 (5-9)
Drug class category, No. (%)		
Antiandrogens/HRAs	70 (38)	59 (29)
Antineoplastic agents	113 (62)	146 (71)
Line of treatment, No. (%)		
1st line	73 (40)	99 (48)
2nd line	51 (28)	52 (25)
3rd line	28 (15)	27 (13)
4th line	31 (17)	27 (13)
Diagnosis category, No. (%)		
Breast	32 (17)	30 (15)
Central nervous system	24 (13)	20 (10)
GI	18 (10)	40 (20)
GU	84 (46)	81 (40)
Other^a^	25 (14)	34 (17)
Cancer stage, No. (%)		
Stage I or II	36 (20)	30 (15)
Stage III	36 (20)	30 (15)
Stage IV	108 (59)	142 (69)
Staging not available	3 (2)	3 (1)
ECOG score, No. (%)		
0	65 (36)	69 (34)
1	80 (43)	93 (45)
2	12 (7)	20 (10)
3	0	2 (1)
Unknown/not documented	26 (14)	21 (10)
Hospitalized within 3 months prior to initial counseling, No. (%)	66 (36)	84 (41)

Abbreviations: CCI, Charlson comorbidity index; ECOG, Eastern Cooperative Oncology Group; GI, gastrointestinal; GU, genitourinary; HRA, hormone receptor antagonist; IQR, interquartile range.

^a^Other diagnoses include gynecologic, head/neck, lung, and melanoma/sarcoma.

### Pharmacist interventions

**Figure 2.**
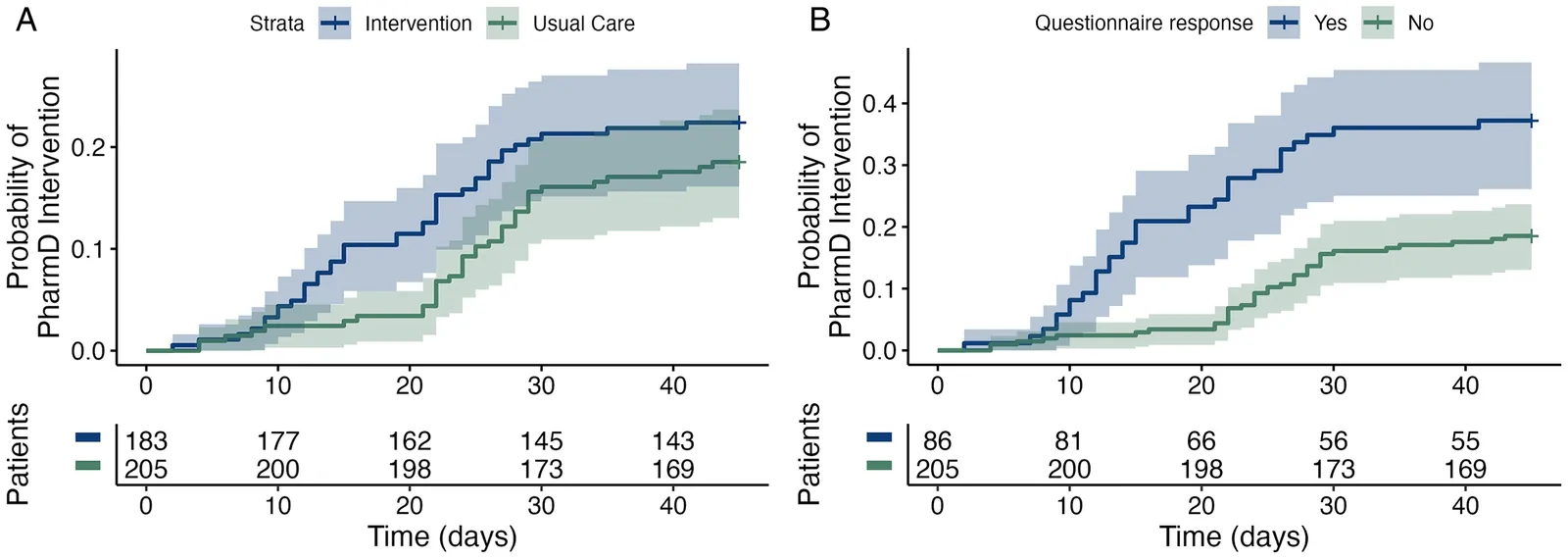
Time to pharmacist intervention in the intention-to-treat (ITT) and per-protocol analyses. In the ITT analysis (A), there was no significant difference in time to first adverse effect (AE)–related intervention between the intervention and usual care cohorts (hazard ratio [HR], 1.2; 95% CI, 0.8-1.9; *P* = 0.360). However, in the per-protocol analysis (B) evaluating questionnaire responders (blue) and patients receiving usual care (green), responders had a significantly faster time to AE-related intervention (HR, 2.4; 95% CI, 1.5-4.0; *P* < 0.001).

**Figure 3.**
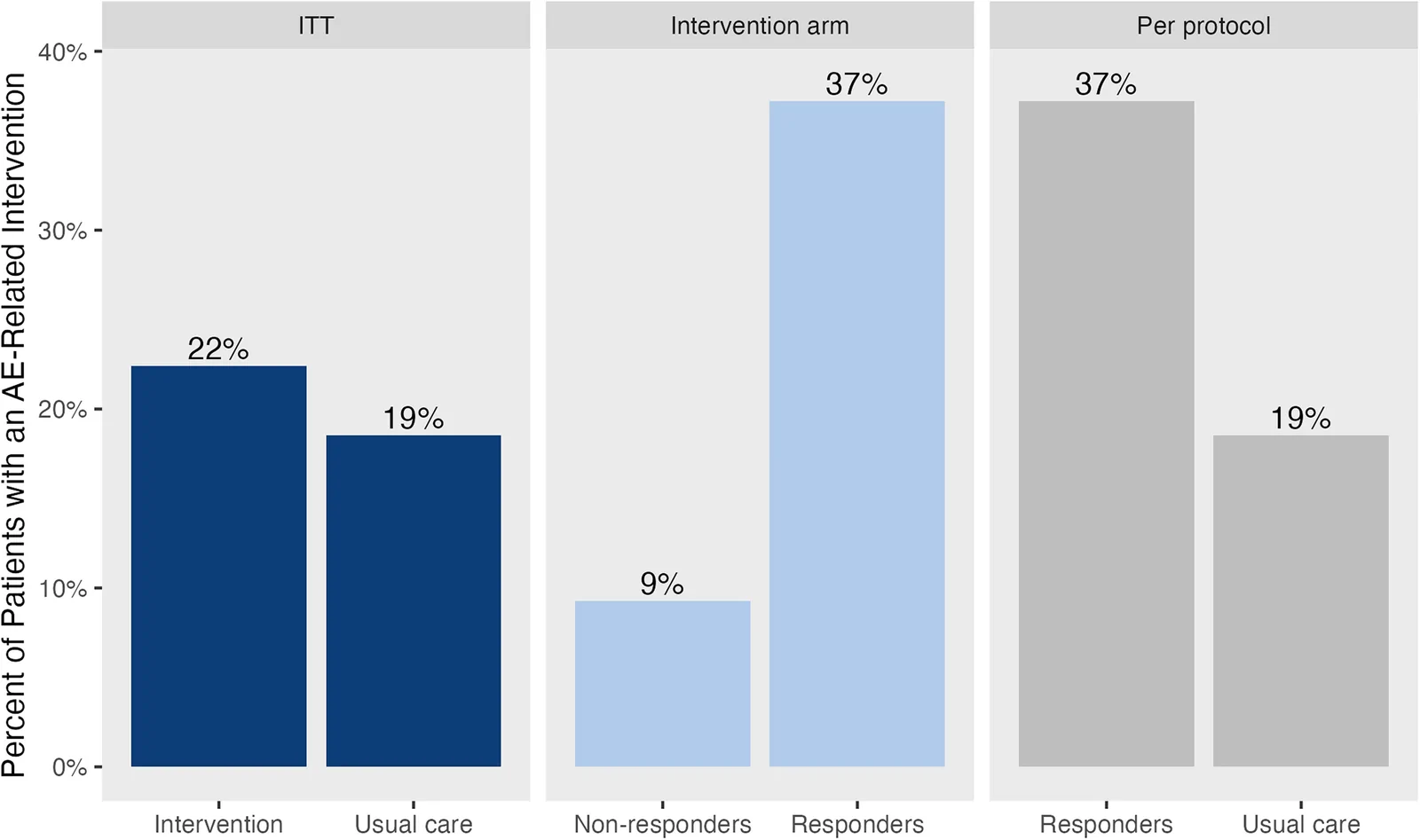
Frequency of adverse event (AE)–related interventions within 45 days of the first oral anticancer medication dispense. In the intention-to-treat (ITT) analysis (dark blue bars), there was no significant difference between the usual care and intervention cohorts. Looking at intervention patients only (light blue bars), a higher percentage of questionnaire responders versus nonresponders had an intervention. In the per-protocol analysis (gray bars), questionnaire responders were significantly more likely than usual care patients to have interventions.

For the ITT analysis (intervention vs usual care), the time to AE-related intervention was not significantly different between the intervention and usual care cohorts (hazard ratio [HR], 1.2; 95% CI, 0.8-1.9; *P* = 0.360) (Figure 2A), nor was the number of pharmacist interventions (odds ratio [OR], 1.2; 95% CI, 0.8-2.0; *P* = 0.409). No other covariates were significant. Roughly a quarter of intervention patients (22%) had at least 1 AE-related intervention in the first 45 days, with a median time to intervention of 19 days (IQR, 12-25 days). A lower proportion of usual care patients (19%) had an AE-related intervention, with a median time to first AE-related intervention of 25 days (IQR, 22-29 days) (Figure 3).

In the PP analysis comparing intervention patients who responded to the eTEAM questionnaire (n = 86) with the usual care cohort (n = 205), intervention patients who responded had a significantly faster time to first pharmacist intervention (HR, 2.4; 95% CI, 1.5-4.0; *P* < 0.001) (Figure 2B) and significantly higher odds of having more interventions (OR, 2.7; 95% CI, 1.5-4.9; *P* < 0.001). The first AE-related intervention occurred at a median of 15 days (IQR, 12-23 days) for intervention responders and a median of 25 days (IQR, 22-29 days) for usual care patients. Additionally, 37% of responders had at least 1 AE-related intervention in the first 45 days, compared to 19% of those in the usual care cohort (Figure 3).

### Interventions performed

**Figure 4.**
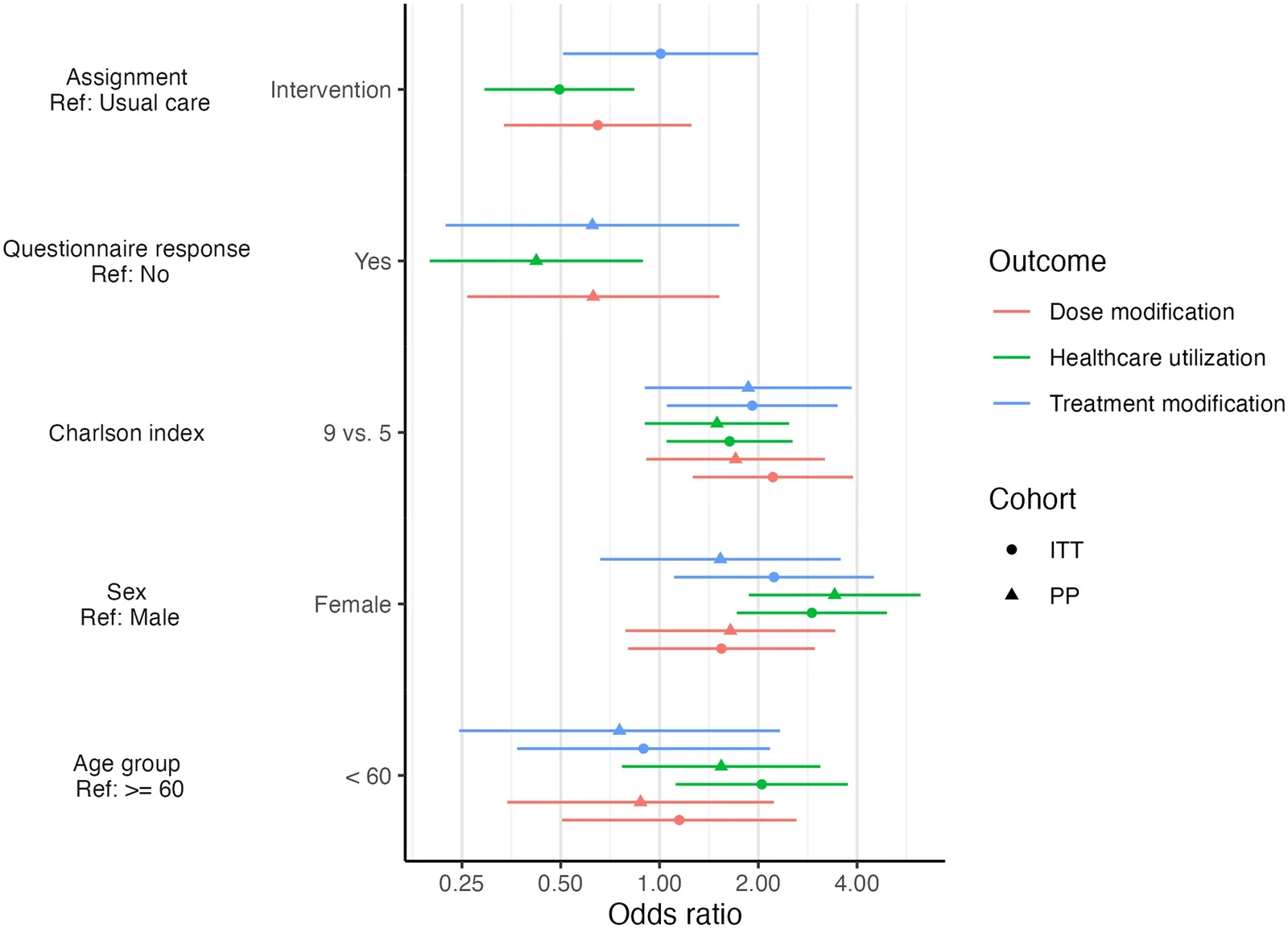
Results for evaluation of (1) dose modifications, (2) healthcare utilization, and (3) treatment modifications within 90 days of initial medication dispensing in the intention-to-treat (ITT) analysis and per-protocol (PP) analysis, as determined using ordinal logistic regression models. Intervention patients (ITT analysis) and intervention responders (PP analysis) were half as likely to have healthcare utilization within 90 days. Though modeled as a per unit change, Charlson comorbidity index is depicted as a comparison of the 75th percentile of the data versus the 25th percentile.

There were a total of 107 AE-related interventions (intervention, n = 59; usual care, n = 48) performed in 79 patients (intervention, n = 41; usual care, n = 38). Table 2 details interventions by cohort. For the entire sample, AE-related interventions involved alerts due to adherence (n = 15, 14%), medication reconciliation/interaction (n = 10, 9%), healthcare use (n = 5, 5%), an administration/dosing issue (n = 5, 5%), and care coordination (n = 3, 3%). Multiple actions could have been performed for a single intervention. Patient chart review(eg, laboratory resultreview, medication reconcilitation, updates to patient profile) (n = 75, 70%) and patient counseling (n = 75, 70%) were the most common actions. Some interventions resulted in adjunct therapy being started (n = 5, 5%) (intervention patients only), follow-up care being scheduled (n = 4, 4%), medication being held (n = 4, 4%), and/or a dose being adjusted (n = 1, 1%) (Figure 4).

**Table 2.** Pharmacist Interventions by Cohort Assignment and Overall

	Study assignment		
	Intervention (n = 59)	Usual care (n = 48)	Overall (N = 107)
**Category of alert**			
Adverse event	59 (100)	48 (100)	107 (100)
Adherence	7 (12)	8 (17)	15 (14)
Medication reconciliation/ interaction	3 (5)	7 (15)	10 (9)
Healthcare use	2 (3)	3 (6)	5 (5)
Administration issue	4 (7)	1 (2)	5 (5)
Care coordination	2 (3)	1 (2)	3 (3)
**Action**			
Patient counseling provided	46 (78)	29 (60)	75 (70)
Patient chart reviewed	35 (59)	40 (83)	75 (70)
Prescriber contacted	8 (14)	7 (15)	15 (14)
Medication reconciliation	1 (2)	5 (10)	6 (6)
Referred to care	1 (2)	0	1 (1)
Financial counseling provided	1 (2)	0	1 (1)
Education provided	1 (2)	0	1 (1)
**Intervention outcome**			
Issue resolved	54 (92)	43 (90)	97 (91)
Adjunct therapy started	5 (8)	0	5 (5)
Medication held	2 (3)	2 (4)	4 (4)
Care scheduled	2 (3)	2 (4)	4 (4)
Dose adjusted	0	1 (2)	1 (1)

### Extended impact on outcomes

Dose modifications occurred in 9% and 14% of intervention and usual care patients, respectively. Treatment modifications occurred in 9% of intervention patients and 10% of usual care patients. Fifteen percent of intervention patients required healthcare utilization, compared to 26% of usual care patients (Table 3). Dose modification, treatment modifications, and healthcare utilization for the PP cohort only are shown in eTable 2.

**Table 3.** Clinical Outcomes at 90 Days by Cohort Assignment and Overall^a^

	Study assignment		
	Intervention (n = 183)	Usual care (n = 205)	Overall (N = 388)
**No. of dose modifications (dose interruptions and dose changes)**			
0	167 (91)	177 (86)	344 (89)
1	13 (7)	19 (9)	32 (8)
2	3 (2)	9 (4)	12 (3)
**No. of healthcare utilization instances (hospitalizations and ED visits)**			
0	156 (85)	151 (74)	307 (79)
1	20 (11)	34 (17)	54 (14)
2	4 (2)	16 (8)	20 (5)
3	1 (1)	4 (2)	5 (1)
4	1 (1)	0	1 (<1)
6	1 (1)	0	1 (<1)
**No. of treatment modifications (discontinuations and medication changes)**			
0	166 (91)	184 (90)	350 (90)
1	7 (4)	14 (7)	21 (5)
2	10 (5)	6 (3)	16 (4)
3	0	1 (<1)	1 (<1)

Abbreviation: ED, emergency department.

^a^All data are No. (%) of pharmacist interventions.

In the ITT analyses, no significant differences were seen in dose (OR, 0.7; 95% CI, 0.3-1.3; *P* = 0.196) or treatment (OR, 1.0; 95% CI, 0.5-2.0; *P* = 0.981) modifications at 90 days between patients in the intervention and usual care cohorts; however, intervention patients were half as likely to have healthcare utilization than usual care patients (OR, 0.5; 95% CI, 0.3-0.8; *P* = 0.009). A higher CCI score was associated with a higher risk of dose modifications (OR per 1 unit increase, 1.2; 95% CI, 1.1-1.4; *P* = 0.006) and treatment modifications (OR, 1.2; 95% CI, 1.01-1.4; *P* = 0.033). Females were also twice as likely to have a treatment modification (OR, 2.2; 95% CI, 1.1-4.5; *P* = 0.025). Patients with a higher CCI (OR, 1.1; 95% CI, 1.01-1.3; *P* = 0.029), females (OR, 2.9; 95% CI, 1.7-4.9; *P* < 0.001), and older patients (OR, 2.0; 95% CI, 1.1-3.7; *P* = 0.020) were more likely to have healthcare utilization (Figure 4).

In the PP analyses, no significant differences were seen in dose (OR, 0.6; 95% CI, 0.3-1.5; *P* = 0.303) or treatment (OR, 0.6; 95% CI, 0.2-1.7; *P* = 0.370) modifications at 90 days for patients in the intervention responders subgroup versus the usual care cohort; however, intervention responders were 60% less likely than usual care patients to have healthcare utilization (OR, 0.4; 95% CI, 0.2-0.9; *P* = 0.023). In the PP analysis, CCI score and age were no longer significantly correlated with dose or treatment modification, but females were still more likely to have healthcare utilization (OR, 3.4; 95% CI, 1.9-6.2; *P* < 0.001) (Figure 4).

## Discussion

### Impact of eTEAM questionnaire on early pharmacist intervention and AE identification

Patients who responded to the eTEAM questionnaire had a faster time to pharmacist intervention, had a significantly higher number of AEs identified, and were 2.7 times more likely to receive pharmacist interventions than those in the usual care cohort. These findings align with previous literature recommending early monitoring within the first 7 to 14 days after initiation of an oral anticancer medication^20^ and confirm other study results showing the benefit of pharmacist-led early check-in protocols in addressing oral anticancer medication–related issues.^34^ Pharmacists commonly counseled patients in response to reported AEs and initiated adjunct (supportive) therapy in some patients to mitigate AEs and support medication persistence. Similar pharmacist intervention results were seen with use of another HSSP-led early check-in protocol.^34^ AEs are common early in cancer treatment, and early identification is important as AEs can impact the ability for patient to remain on therapy.^35^ These results confirm previous studies identifying the value of pharmacists in oral chemotherapy management and AE mitigation^36^ and validate that the eTEAM questionnaire is an effective tool at identifying areas for pharmacist intervention. Notably, all patients included in the study had an active patient portal and the direct phone number to the pharmacist, providing an opportunity to report AEs or adherence issues to the healthcare team if they were motivated to do so, a more passive approach to monitoring. The eTEAM questionnaire was a proactive approach by the healthcare team to check in on patients rather than relying on them to contact the healthcare team, which resulted in a higher rate of identifying AE-related concerns.

### Impact of eTEAM questionnaire on healthcare utilization

Our results show that patients in the usual care cohort less commonly had adjunct therapy initiated for AE mitigation (intervention, n = 5; usual care, n = 0) and more frequently used additional healthcare than those in the intervention cohort (n = 54 [26%] vs n = 27 [15%]). During AE-related interventions, pharmacists provide AE mitigation strategies, educate on the timing and duration of AEs, make recommendations for dose adjustments or holds, and facilitate coordination of care. These findings suggest that pharmacist outreach to patients soon after initiation of oral anticancer treatment results in the opportunity to manage AEs earlier, which may reduce the need for patients to pursue additional healthcare resources such as hospitalizations and ER visits to treat AEs that have escalated in severity. Brands et al^37^ also found that use of patient-centered digital health records that provide access to e-health tools such as patient portals may result in fewer ER visits and hospitalizations. AE-related healthcare costs are substantial and contribute to the economic burden of cancer care.^38^ Proactive AE management is shown to reduce costs related to ER visits and hospitalizations.^39^ These results further demonstrate that the timing of monitoring, particularly in patients starting oral anticancer medications, may also impact healthcare utilization rates.

Age, CCI score, and sex were significantly associated with healthcare utilization at 90 days in the ITT analysis, and sex remained significant in the PP analysis. It is not surprising that patients with older age and more comorbidities were more likely to require additional healthcare utilization. Additionally, existing literature shows that females tend to utilize healthcare services more than men, which supports the findings observed in this study. Larger studies are needed to validate these findings.

### Patient response to eTEAM questionnaire

Notably, the PP analysis found that in patients who received a questionnaire but did not respond (around half), there was not a significant difference in time to intervention and the rate of AEs identified when those patients were compared to patients who responded to the questionnaire. Simply implementing the questionnaire did not result in a significant outcome, which emphasizes the importance of patient engagement in early monitoring programs. Patients’ response to the questionnaire indicated a higher level of active engagement, allowing for prompt identification of issues. However, patients who did not respond had outcomes similar to those receiving usual care. Barriers to patient engagement with EHR portals, such as health literacy, digital confidence, and patient preference, have been noted in previous research and should be considered.^23^ The relatively high questionnaire response rate in this study highlights that patients will use this medium for reporting clinical information and providing patients with multiple mediums that fit their engagement preferences is useful.

### Impact of eTEAM questionnaire on clinical workflow

Previous studies have demonstrated the benefit of early monitoring for patients initiated on treatment with oral anticancer medications,^34,40^ but there is minimal literature describing how to implement monitoring, particularly addressing the increase in workload. This study sought to offer a tool for monitoring that did not overburden clinic staff. To keep clinic workflow disruption to a minimum and not influence the patient response rate, pharmacists did not follow up with patients if there was not a response to the initial questionnaire. In the previously published report on the implementation phase,^28^ pharmacists noted that incorporating electronic monitoring into the workflow was not as burdensome as initially perceived and felt that patient care was improved with electronic early monitoring.^28^ Our results demonstrate that using the patient portal within the EHR for early monitoring can help identify AEs earlier in treatment, allow for early pharmacist intervention, and achieve early monitoring goals without being overly burdensome for clinic pharmacists. Directives to increase patient response, such as re-sending the questionnaire or reaching out to the patient via phone correspondence, can be considered.

### Contribution to current literature

Zerillo et al^41^ described the lack of literature and limitations in study design regarding best practices for management of patients on oral anticancer medications. A strength of our study was the use of a randomization tool within the EHR developed by the institution’s health IT department to compare the intervention versus usual care cohorts in a real-world, prospective setting. This design allows for statistical analysis of patient outcomes that is missing from other early monitoring studies^34^ and adds to the small volume of existing work demonstrating improvement in patient clinical end points as a result of patient monitoring.^42^

### Limitations

Limitations of the study include its being conducted at a single center with a relatively small sample size. A large number of patients had a diagnosis of prostate cancer, potentially skewing the applicability to other patient populations. Additionally, our patient population had high EHR utilization at baseline, and generalizability to other institutions where EHR patient portal use is less common may be limited. Patients prescribed medication that followed a tailored monitoring protocol used by the integrated pharmacy were excluded, though this was a small number (approximately 9 patients/month) and was not expected to impact the study results. Automated determination of the patient start date proved to be a challenge, requiring pharmacist chart review to ensure that the patient had initiated treatment on or around the time of initial counseling, as sending the eTEAM questionnaire either too early or too late would not be in line with monitoring recommendations. To address this challenge, pharmacists have started documenting the anticipated start date in a flowsheet within the EHR, allowing for automated release of the questionnaire to the patient. Efforts to build the randomization tool and the patient questionnaire within the EHR required frequent communication and support from the health IT department. Access to these resources may not be available at all practices or health systems, which could be a limitation to implementation.

## Conclusion

Using EHR questionnaires to monitor patients initiated on oral anticancer medications can improve early AE identification, allowing pharmacists to provide meaningful interventions and potentially reducing healthcare utilization.

## Supplementary Material

zxag070_Supplementary_Data

## Data Availability

The data underlying this article cannot be shared publicly due to the nature of the research and ethical and privacy concerns. Select deidentified data elements may be shared upon request to the corresponding author.
