# Supplementary material for: The impact of early oral anticancer medication monitoring through electronic patient assessments
Source: Am J Health Syst Pharm. 2026 Mar 7;83(17):e637–48. doi: 10.1093/ajhp/zxag070 (PMC13487789; doi:10.1093/ajhp/zxag070)
Supplement: Supplementary file 1 [file zxag070_supplementary_data.zip › -2025AJHP0491-file005.docx]

**eTable 1**. Demographic and Clinical Data for Study Cohorts

| **Characteristic** | **Overall**  N = 388 | **Intervention**  N = 183 | **Usual Care**  N = 205 | **Questionnaire responders**^b^  N = 86 |
| --- | --- | --- | --- | --- |
| **Sex: Male, n (%)** | 228 (59) | 112 (61) | 116 (57) | 60 (70) |
| **Age group: >= 60, n (%)** | 268 (69) | 124 (68) | 144 (70) | 62 (72) |
| CCI, Median (IQR) | 7 (5 - 9) | 7 (5 - 9) | 8 (5 - 9) | 7 (5 - 9) |
| **Drug class category, n (%)** |  |  |  |  |
| Anti-androgens/hormone receptor antagonists | 129 (33) | 70 (38) | 59 (29) | 44 (51) |
| Anti-neoplastic agents | 259 (67) | 113 (62) | 146 (71) | 42 (49) |
| **Line of Treatment, n (%)** |  |  |  |  |
| 1st line | 172 (44) | 73 (40) | 99 (48) | 45 (52) |
| 2nd line | 103 (27) | 51 (28) | 52 (25) | 22 (26) |
| 3rd line | 55 (14) | 28 (15) | 27 (13) | 10 (12) |
| 4th line or higher | 58 (15) | 31 (17) | 27 (13) | 9 (10) |
| **Diagnosis category, n (%)** |  |  |  |  |
| Breast | 62 (16) | 32 (17) | 30 (15) | 11 (13) |
| Central nervous system | 44 (11) | 24 (13) | 20 (10) | 10 (12) |
| GI | 58 (15) | 18 (10) | 40 (20) | 7 (8) |
| GU | 165 (43) | 84 (46) | 81 (40) | 48 (56) |
| Other | 59 (15) | 25 (14) | 34 (17) | 10 (12) |
| **Cancer stage, n (%)** |  |  |  |  |
| stage I | 17 (4) | 8 (4) | 9 (4) | 2 (2) |
| stage II | 49 (13) | 28 (15) | 21 (10) | 18 (21) |
| stage III | 66 (17) | 36 (20) | 30 (15) | 16 (19) |
| stage IV | 250 (64) | 108 (59) | 142 (69) | 48 (56) |
| staging not available | 6 (2) | 3 (2) | 3 (1) | 2 (2) |
| **ECOG score, n (%)** |  |  |  |  |
| 0 | 134 (35) | 65 (36) | 69 (34) | 34 (40) |
| 1 | 173 (45) | 80 (44) | 93 (45) | 36 (42) |
| 2 | 32 (8) | 12 (7) | 20 (10) | 2 (2) |
| 3 | 2 (1) | 0 (0) | 2 (1) | 0 (0) |
| Unknown or not documented | 47 (12) | 26 (14) | 21 (10) | 14 (16) |
| **Hospitalized within 3 months prior to initial counseling, n (%)** | 150 (39) | 66 (36) | 84 (41) | 26 (30) |

Abbreviations. CCI: Charlson Comorbidity Index; IQR: interquartile range, HRAs: hormone receptor antagonists; GI: gastrointestinal; GU: genitourinary; ECOG: Eastern Cooperative Oncology Group

a: Other diagnoses include: GYN, Head/Neck, Lung, Melanoma/sarcoma

b: Questionnaire responders are a subgroup of the intention-to-treat population, consisting of patients who responded to the survey questionnaires.
