# Supplementary material for: The impact of early oral anticancer medication monitoring through electronic patient assessments
Source: Am J Health Syst Pharm. 2026 Mar 7;83(17):e637–48. doi: 10.1093/ajhp/zxag070 (PMC13487789; doi:10.1093/ajhp/zxag070)
Supplement: Supplementary file 1 [file zxag070_supplementary_data.zip › -2025AJHP0491-file006.docx]

eTable 2. PP analysis of Clinical Outcomes at 90 Days

|  | **Study assignment** | |  |
| --- | --- | --- | --- |
| **Characteristic, n (%)** | **Questionnaire**  **responders**  N = 86 | **Usual care**  N = 205 | **Overall**  N = 291 |
| **Number of dose modifications**  **(Dose interruptions and dose changes)** |  |  |  |
| 0 | 79 (92) | 177 (86) | 256 (88) |
| 1 | 6 (7) | 19 (9) | 25 (9) |
| 2 | 1 (1) | 9 (4) | 10 (3) |
| **Number of healthcare utilization instances (Hospitalizations and ED visits)** |  |  |  |
| 0 | 76 (88) | 151 (74) | 227 (78) |
| 1 | 9 (10) | 34 (17) | 43 (15) |
| 2 | 1 (1) | 16 (8) | 17 (6) |
| 3 | 0 (0) | 4 (2) | 4 (1) |
| **Number of treatment modifications (Discontinuations and medication changes)** |  |  |  |
| 0 | 81 (94) | 184 (90) | 265 (91) |
| 1 | 2 (2) | 14 (7) | 16 (5) |
| 2 | 3 (3) | 6 (3) | 9 (3) |
| 3 | 0 (0) | 1 (0) | 1 (0) |
